# COVID-19 and Stressful Adjustment to Work: A Long-Term Prospective Study About Homeworking for Bank Employees in Italy

**DOI:** 10.3389/fpsyg.2022.843095

**Published:** 2022-03-17

**Authors:** Maria Donata Orfei, Desirée Estela Porcari, Sonia D’Arcangelo, Francesca Maggi, Dario Russignaga, Nicola Lattanzi, Andrea Patricelli Malizia, Emiliano Ricciardi

**Affiliations:** ^1^Molecular Mind Laboratory (MoMiLab), IMT School for Advanced Studies Lucca, Lucca, Italy; ^2^Neuroscience Lab, Intesa Sanpaolo Innovation Center S.p.A., Turin, Italy; ^3^Intesa Sanpaolo DC Tutela Aziendale - Sicurezza sul Lavoro ed Ambiente, Turin, Italy; ^4^Laboratory for the Analysis of Complex Economic Systems (AXES), IMT School for Advanced Studies Lucca, Lucca, Italy

**Keywords:** employees, work-family conflict, COVID-19, pandemic, stress, homeworking

## Abstract

The COVID-19 evolution has forced the massive introduction of homeworking (HW) for most employees in the initial stages of the pandemic and then return to work, mainly due to the vaccination campaign. These multiple abrupt adjustment demands in work may be a source of intense stress for office workers with consequences on wellbeing and the quality of life. This long-term prospective study aimed at investigating the effect of adaptation demands on a broad population of employees of a large Italian banking group in the job-related stress framework. We administered a web-based survey to 1,264 participants in Reopening after the first lockdown, from June to October 2020, at 841 subjects in Second Wave, corresponding to the rise of contagions from November 2020 to January 2021, and to 491 individuals in Vaccination Round, which ranged from February to June 2021. We assessed workaholism by using the Dutch Work Addiction Scale (DUWAS-10), work-family conflicting overlap by using the Work and Family Conflict Scale (WAFCS), and concern for back to work (BW) and for HW by specific questions. Higher WAFCS scores characterized Reopening and Vaccination Round while Second Wave had the highest level of concern for HW. Women and younger individuals showed the highest concern for BW, WAFCS, and DUWAS-10 scores regardless of the pandemic stage. HW days per week were related to more heightened concern for BW and lower concern for HW, DUWAS, and WAFCS scores. The number of children was related to lower Concern for BW and higher WAFCS scores in Reopening and Second Wave. Our data showed that massive adjustment demands in work and family routine represented a significant source of stress for employees, regardless of the different pandemic stages. The highest level of fatigue emerged in women and younger subjects. These results shed light on the need for a road map to promote a gradual and structured adjustment for workers and encourage organizations to consider homeworking as a valid stable alternative.

## Introduction

In the present pandemic context, the extent to which employees can adjust to work changes is crucial for individual and organizational outcomes (van Zoonen et al., 2021). The job framework has undergone massive changes in a few months. In Italy, homeworking (HW) has been vigorously promoted as a COVID-19 containment measure worldwide since the outbreak of the pandemic. Most public and private companies employees started to work from home 5 days a week, although in some cases, particularly after the end of the lockdown, the days spent in presence at the workplace could be variable. Consequently, a forced revolution has taken place in a few weeks, thus compelling a strenuous effort to adjust. Subsequently, the relatively rapid changes in the socio-sanitary situation, particularly the implementation of the vaccination campaign, have recently encouraged a progressive return to the workplace.

### Stressful Challenges in the Pandemic Job Context

According to classical stress theories, these abrupt changes in the job context may determine relevant distress in office workers. First, office workers accumulated a massive adaptive effort (Holmes and Rahe, 1967; Cohen et al., 2019) at the beginning of the pandemic events to convert their usual office routine into HW and, more recently, to return to office routine again. Second, in the pandemic context, HW was not a free choice for employees but a forced solution. Thus, control over job situations and predictability were very low (Karasek, 1971; Karasek et al., 1981), and the worker could directly take no action or choice. Finally, turning to HW may have interrupted or altered an individual’s career primary goals, and expectations (Carver and Scheier, 1999), as well as going back to the workplace may be perceived as an interruption of one’s goals to maintain the physical integrity and psychological wellbeing (Lazarus and Folkman, 1984; Kemeny, 2003).

Given these premises, workers could be susceptible to developing three specific stress-related phenomena threatening the quality of life and mental health: workaholism, family-work overlap, and skepticism about back to work (BW).

### Workaholism

Prolonged and forced HW, along with the stressful perception of threats to one’s career, can solicit the shift from work engagement to workaholism (Shimazu et al., 2015; Di Stefano and Gaudiino, 2018). Workaholism is characterized by excessive work involvement, drive, poor enjoyment, the feeling that one cannot stop job activities, a high sense of pressure, and distress (Spence and Robbins, 1992). Indeed, HW can imply a dramatic increase in the number of hours spent at a workstation, which in turn is associated with worse mental health (DeFilippis et al., 2020; Awada et al., 2021; Magnavita et al., 2021), and determine lower engagement and motivation (Canonico, 2016). Finally, the massive use of technology during HW and the deriving technostress (Salanova et al., 2013), have shown to be related to worse mental health and workaholism (Molino et al., 2020; Spagnoli et al., 2020). To note, the sudden change in working processes might have been particularly detrimental for workers who were previously addicted to their job, as they could perceive the change as upsetting their usual job routine, with an amplified feeling of anxiety and frustration, and, therefore, in general, as a more stressful experience (Spagnoli et al., 2020). In addition, a recent study cast light on the role of job control as defined by decision-making autonomy in modulating workaholism and emotional exhaustion (Spagnoli and Molinaro, 2020). Far from being a trivial issue, workaholism deserves attention as it is considered a predictor of anxiety and depression (Serrano-Fernández et al., 2021), physical health problems and strained social relationships, and poor job performance in the long run (Ng et al., 2007; Clark et al., 2016).

### Family Life and Work Demands Overlap

A second issue related to the stressful effects of sudden and repeated changes in job context concerns is the overlap between family life and work activity, which may not be easy to handle. HW may facilitate caring for children and sick relatives (Moretti et al., 2020) but can also show the dark side of burnout (Barriga Medina et al., 2021). The main effect of forced HW is a role conflict, as the simultaneous presence of two sources of pressure, that is, work and family may lead to a stressful dilemma due to the necessity to fulfill both work role and family role, at the same time, in the same place (Fukumura et al., 2021; Dabbagh et al., 2022). Additionally, the altered working hours in HW may have a detrimental effect on parental functioning, exacerbating psychological distress, work-family conflict, and relationship quality (Zhao et al., 2021). Possibly, women suffered blurred work-life boundaries, with more severe difficulties in balancing work schedules around other family members so that work time may have become “porous” (Genin, 2016; Xiao et al., 2021).

### Back to Work vs. Homeworking Dilemma

Finally, an additional source of stress is the recent encouragement to go BW. In addition to the previous stressful phenomena, returning to traditional work contexts giving up HW may raise skepticism in employees. This attitude may be due to the conflict between the pressure to keep the job, the eagerness to return to normal life, and the fear of contamination. Thus, returning to work may not be an easy decision for employees, primarily when it occurs in an uncertain environment (Balkhi et al., 2020; Shaw et al., 2020). Furthermore, BW is still far from previous “normality,” as some precautional measures, such as physical distancing or wearing face masks in the workplace, have still to be granted that may harm the workers’ mental wellbeing (Hamouche, 2021). Finally, the prolonged condition of HW, along with the experience of possible economic and organizational advantages, may have brought new adjustments in family organizations, which can be hard to give up for workers.

### Aims and Hypotheses of the Study

The articles’ mixed results and varying quality create challenges in drawing out meaningful knowledge on the impacts of continuous adjustment to the changing rules and safety conditions on the individuals’ mental health (Oakman et al., 2020).

Our study aimed at investigating the psychological adaptation to sudden changes in the work context in a large sample of Italian employees in a long-term perspective. For this, we have developed a three-survey case study comparing three different pandemic stages, namely: the Reopening after the end of the first lockdown of March 2020, the Second Wave in October and November 2020, and the first Vaccination Round in May and June 2021. Thus, overall, we considered the pandemic period from June 2020 to June 2021, focusing on specific organizational change periods. In particular, we analyzed (a) the level of workaholism and work-family interference, (b) concern about BW and HW, and (c) the socio-demographic predictors for psychological adjustment to HW. We have administered an online survey at three different time -points, such as socio-demographic questions and structured questionnaires.

We expected that: (a) the Reopening and the Vaccination Round, as a moment of forced changes in the work routine, would be characterized by the higher levels of workaholism and work-family conflict; (b) since worldwide being a woman and lower age represented risk factors for developing psychological symptoms, such as anxiety, depression, and sleep disorders, due to the impact of the pandemic even in longitudinal studies (Gualano et al., 2020; Prati, 2020; Amicucci et al., 2021; Marelli et al., 2021; Salfi et al., 2021a), women and younger subjects would show the higher levels of workaholism and work-family interference, regardless of the time point of assessment; (c) higher Concern about BW and higher work-family conflict would be related to the higher number of children in a family and the higher number of days spent in HW.

## Materials and Methods

### Study Design

A three-survey case study design was adopted. All participants were provided with a detailed description of the experimental procedures and required consent before participating in the study. The survey was anonymous, as each participant was assigned an alphanumeric code so that the confidentiality of information was assured. Data were collected three times: from September 21 to October 11, 2020 (Reopening), from January 11 to January 31, 2021 (Second Wave), and from May 24 to June 4, 2021 (Vaccination Round). Each subject could fill out the questionnaire only once, although participants could terminate the survey within a week. Questionnaires were evenly distributed across the national territory. The Reopening survey included questions that referred explicitly to the period from June 1 to October 10, 2020, corresponding in Italy to the months immediately after the first lockdown. The Second Wave survey included questions that referred to November 2020 to January 2021, fitting to the second pandemic wave in Italy. The Vaccination Round survey included questions from February to June 2020, corresponding to the first vaccination campaign in Italy. The study was conducted in accordance with the ethical standards laid down in the 1964 Declaration of Helsinki and under a protocol approved by the Joint Ethical Committee for Research of Scuola Normale Superiore, Scuola Superiore Sant’Anna, and IMT School for Advanced Studies Lucca (protocol 04/2021).

### Participants

A panel of 3,000 employees of a large Italian banking group was invited to participate in an online survey. The same panel was invited for each of the three surveys. In this banking group, most employees worked at home during the first year of the pandemic, although an alternating model HW and working at the office was encouraged progressively. At any time point, inclusion criteria were: (a) age higher or equal to 18 years old, (b) Italian mother tongue or high-level knowledge of Italian language, and (c) living in Italy since the pandemic outbreak (i.e., from March 2020).

### Assessment

#### Socio-Demographic Data

The survey included gender, age, number of days spent in HW, and number of children in a family at each time point. According to the literature, we categorized age into three groups (under 35 years old, 36–54 years old, and over 55 years old) (De Rosa et al., 2014; ISTAT, 2020; OECD, 2021).

#### The Dutch Work Addiction Scale (DUWAS-10)

The abbreviated version of the Dutch Work Addiction Scale (DUWAS) (Schaufeli et al., 2009) was used to assess workaholism. It is a 10-item self-administered questionnaire developed according to Schaufeli et al. (2009) definition of workaholism as “the tendency to work excessively hard (behavioral dimension) and being obsessed with work (cognitive dimension), which manifests itself in working compulsively.”. The two-factor structure of this scale, Working Excessively and Working Compulsively, measured with five items each, has been confirmed across different populations (Del Libano et al., 2010; Rantanen et al., 2015). An Italian version of the questionnaire (Nonnis et al., 2017) has been used in this study.

#### The Work and Family Conflict Scale

The possible conflicting overlap between family and work demands was investigated by the Work and Family Conflict Scale (WAFCS) (Netemeyer et al., 1996; Colombo and Ghislieri, 2008). The questionnaire includes two independent subscales: the work-family conflict, investigating the perceived detrimental interference of work demands on family life (e.g., “The demands of my work interfere with my home and family life”), and the work-family conflict, assessing the family issues that prevent a satisfying job performance (e.g., “I have to put off doing things at work because of demands on my time at home”). Specifically, the two subscales highlight the conflict between the role responsibilities of the two life domains and the extent to which they are incompatible. Each subscale consists of 5 items, each rated on a 7-point Likert scale (1 = strongly disagree and 7 = strongly agree).

#### Concern About Back to Work and Homeworking

To investigate the Concern about BW and HW, we asked two questions: “How concerned are you about going back to the office?” and “How concerned are you about working at home?,” respectively. Each item was rated on a 5-point Likert scale (1 = no concerned at all and 5 = extremely concerned”).

### Statistical Analyses

Statistical analyses were performed using IBM SPSS software ver. 23.

Comparisons among Reopening, Second wave, and Vaccination Round groups on gender and age classes were made using the chi-square test; the same statistical procedure was used to perform comparisons between age classes on gender at each time point. For comparisons on continuous variables, such as the number of children and the number of HW days per week, was used the Kruskal–Wallis test.

We performed a one-way analysis of covariance (ANCOVA) followed by Bonferroni’s *post hoc* tests to compare continuous variables, namely Concern about BW, Concern about HW, and WAFCS at Reopening, Second Wave, and Vaccination Round controlling for gender, age classes, number of children, and the number of HW days per week. The same statistical procedure was used to compare continuous variables, such as DUWAS sub-scores, between the three different age classes at each time point controlling for gender. Independent-samples *t*-tests were used to compare these continuous variables between women and men. Since we did not assess workaholism in Vaccination Round, an independent-samples *t*-test was conducted to compare DUWAS sub-scores between the Reopening and Second Wave groups.

In addition, Pearson’s correlation analyses were carried out to detect the possible associations among DUWAS sub-scores, WAFCS sub-scores, Concerns about BW and HW levels, and the number of children and number of HW days/week in Reopening, Second Wave, and Vaccination Round. A linear regression analysis was performed to highlight predictors.

The significance of all analyses was set at *p* < 0.05 and compensated by the Bonferroni correction for multiple comparisons among total scores.

## Results

At the Reopening survey, 1,264 valid answers were collected; in the Second Wave survey, 841, and in the Vaccination Round survey, 491. The socio-demographic characteristics of the three samples are described in Table 1. The three groups significantly differed on gender and age classes proportion, the number of children, and the number of days per week spent in HW. Since in the Vaccination Round survey, only 37 persons, most of whom were men, provided valid answers at the DUWAS, we chose not to include these data in the analyses to avoid biases in interpreting results. Thus, data for the DUWAS are available only for the Reopening and Second Wave stages. Furthermore, the comparisons between age classes at each time point showed a significant difference in gender proportion.

**TABLE 1:** Demographic characteristics in Reopening, Second Wave, and Vaccination Round groups.

Variable	Reopening *N* = 1264	Second Wave *N* = 841	Vaccination Round *N* = 491		
			
	*N* (%)	*N* (%)	*N* (%)	χ^2^	*P*
Gender				2606	**<0.0001**
Males	813 (64.3)	522 (62.1)	278 (56.6)		
Females	451 (35.7)	319 (37.9)	213 (43.4)		
Age class				2630	**<0.0001**
<35	316 (25)	204 (24.3)	66 (13.4)		
36–54	673 (53.2)	437 (52)	316 (64.4)		
>55	275 (21.8)	200 (23.8)	109 (22.2)		
	***M* ± *SD***	***M* ± *SD***	***M* ± *SD***		
Number of children	1.02 ± 0.9	0.99 ± 0.9	1.2 ± 0.9	272.867	**0.001**
Number of HM days/week	4.45 ± 0.9	4.78 ± 0.7	3.98 ± 1.3	15.033	**<0.0001**
	**Reopening**				
	**<35 *N* = 316**	**36–54 *N* = 673**	**>55 *N* = 275**		
	***N* (%)**	***N* (%)**	***N* (%)**		
Gender				45.172	**<0.001**
Males	165 (52)	431 (64)	217 (79)		
Females	151 (48)	242 (36)	58 (21)		
	**Second Wave**				
	**<35 *N* = 204**	**36–54 *N* = 437**	**>55 *N* = 200**		
	***N* (%)**	***N* (%)**	***N* (%)**		
Gender				35.541	**<0.001**
Males	105 (51)	259 (59)	158 (79)		
Females	99 (49)	178 (41)	42 (21)		
	**Vaccination Round**				
	**<35 *N* = 66**	**36–54 *N* = 316**	**>55 *N* = 109**		
	**N (%)**	**N (%)**	**N (%)**		
Gender				8.304	**0.016**
Males	30 (45)	175 (55)	73 (67)		
Females	36 (55)	141 (45)	36 (33)		

*M, mean; SD, standard deviation; HM, homeworking.*

*Significant values (p < 0.05) are highlighted in bold.*

### Concern About Back to Work and Homeworking, Work and Family Conflict Scale, and Dutch Work Addiction Scale Variables in Reopening, Second Wave, and Vaccination Round

One-way ANCOVA showed that Concern about HW levels was significantly different in the three groups (*F*_2.2593_, 4.411; *p* = 0.012) while controlling for gender, age classes proportion, the number of children, and the number of days per week spent in HW (covariates); Bonferroni’s *post hoc* highlighted that there was a statistically significant difference between Second Wave and Vaccination Round (*p* = 0.009), stressing a higher level in Second Wave group than in the Vaccination Round group.

In the Work and Family Conflict Scale, the work-family conflict level was significantly different in the three groups (*F*_2.2593_, 12.069; *p* < 0.001) while controlling for covariates. Bonferroni test for multiple comparisons highlighted a statistically significant difference between the Reopening and the Second Wave groups (*p* < 0.001), stressing a higher level in the Reopening group than in the Second Wave group.

There was a statistically significant difference in WAFCS family-work conflict in the three groups (*F*_2.2593_, 8.411; *p* < 0.001) while controlling for covariates. Bonferroni’s *post hoc* test highlighted that there was a statistically significant difference between the Reopening and the Second Wave groups (*p* = 0.001) and between the Reopening and the Vaccination Round groups (*p* = 0.008), stressing a higher level in the Reopening group than in the Second Wave and the Vaccination Round groups.

There was no statistically significant difference in Concern about BW and no DUWAS sub-scores emerged (as shown in Table 2).

**TABLE 2:** Questionnaires scores in Reopening, Second Wave, and Vaccination Round groups.

Variable	Reopening *N* = 1264	Second Wave *N* = 841	Vaccination Round *N* = 491	*F*	df	*p*	Reopening vs.		Reopening vs.		Second Wave vs.	
							Second Wave		Vaccination Round		Vaccination Round	
									
							Crit. diff. (or t-test)	*P*	Crit. diff.	*p*	Crit. diff.	*p*
Concern about BW (mean ± *SD*)	2.7 ± 1.2	2,81 ± 1.3	2.53 ± 1.3	0.682	2, 2593	0.506	0.020	1.000	0.077	0.729	0.057	1.000
Concern about HW (mean ± *SD*)	1.82 ± 1.0	1,79 ± 1.0	1.78 ± 1.0	4.411	2, 2593	0.012	−0.055	0.674	0.120	0.079	0.175	0.009
WAFCS Work-family (mean ± *SD*)	13.84 ± 5.1	12.59 ± 5.4	13.42 ± 5.5	12.069	2, 2593	<0.001	1.162	<0.001	0.541	0.167	−0.621	0.136
WAFCS family-work (mean ± *SD*)	8.38 ± 3.7	7.74 ± 3.5	7.91 ± 3.4	8.411	2, 2593	<0.001	0.579	0.001	0.580	0.008	0.001	1.000
DUWAS work- excessively (mean ± *SD*)	11.12 ± 2.9	11.13 ± 2.9	–	0.176	2103	0.674	−0.097	0.923	–	–	–	–
Duwas work- compulsively (mean ± *SD*)	9.72 ± 2.7	9.81 ± 2.8	–	1.163	2103	0.281	−0.073	0.467	–	–	–	–

*BW, back to work; HW, homeworking; WAFCS, work and family conflict scale; DUWAS, Dutch Work Addiction Scale; SD, standard deviation; df, degrees of freedom; crit.diff., critical difference.*

*Significant values (p < 0.05) are highlighted in bold.*

### Concern About Back to Work and Homeworking, Work and Family Conflict Scale, and Dutch Work Addiction Scale Variables in the Reopening Group by Gender and Age Classes

One-way ANCOVA showed a statistically significant difference in Concern about BW between age classes (*F*_2.1261_, 6.267, *p* = 0.002) while controlling for gender (covariate); Bonferroni’s *post hoc* highlighted that there was a significant difference between under 35 and 36–54 groups (*p* = 0.001), stressing a higher level in under 35 groups.

A statistically significant difference in the WAFCS’s work-family conflict sub-scale (*F*_2.1261_, 12.347, *p* < 0.001) and WAFCS family-work conflict sub-scale (*F*_2.1261_, 17.263, *p* < 0.001) emerged between the three age classes while controlling for gender.

Bonferroni’s *post hoc* test highlighted that the WAFCS work-family conflict score was significantly different between under 35 and 36–54 groups (*p* = 0.005) and between 36–54 and over 55 groups (*p* < 0.001); these results stressed a higher level in 36–54 years old subjects. Concerning the WAFCS family work conflict, Bonferroni’s *post hoc* test highlighted significant differences between under 35 and 36–54 groups (*p* < 0.001) and between 36–54 and over 55 (*p* < 0.001). These results stressed a higher level in 36–54 years old subjects.

A statistically significant difference in DUWAS work excessively sub-scale (*F*_2.1261_, 3.558, *p* = 0.029) and DUWAS work-compulsively sub-scale (*F*_2.1261_, 9.654, *p* < 0.001) emerged between the three age classes while controlling for gender. Bonferroni’s *post hoc* test highlighted that the DUWAS work excessively score was significantly different between 36–54 and over 55 groups (*p* = 0.032), stressing a higher level in 36–54 years old subjects. Concerning the DUWAS work-compulsively score, it was significantly different between under 35 and 36–54 groups (*p* = 0.004) and between under 35 and over 55 groups (*p* < 0.001), pointing out a higher level in under 35 subjects.

No statistically significant difference in Concern about HW emerged (as shown in Table 3).

**TABLE 3:** Questionnaires scores in the Reopening group by age class.

Variable	<35 *N* = 316	36–54 *N* = 673	>55 *N* = 275	*F*	df	*p*	<35 vs. 36–54		<35 vs. >55		36–54 vs. >55	
									
							Crit. diff.	*p*	Crit. diff.	*p*	Crit. diff.	*p*
Concern about BW (mean ± *SD*)	2.94 ± 1.3	2,62 ± 1.2	2.65 ± 1.3	6.267	2, 1261	**0.002**	0.296	**0.001**	0.228	0.080	−0.068	1.000
Concern about HW (mean ± *SD*)	1.84 ± 1.0	1,84 ± 1.0	1.75 ± 0.9	0.656	2, 1261	0.519	–	–	–	–	–	–
WAFCS work-family (mean ± *SD*)	13.45 ± 5.1	14.48 ± 5.0	12.72 ± 5.0	12.347	2, 1261	**<0.001**	−1.098	**0.005**	0.569	0.540	1.667	**<0.001**
WAFCS family-work (mean ± *SD*)	7.66 ± 3.1	8.96 ± 3.9	7.82 ± 3.4	17.263	2, 1261	**<0.001**	−1.272	**<0.001**	−0.104	1.000	1.169	**<0.001**
DUWAS work- excessively (mean ± *SD*)	11.08 ± 2.7	11.31 ± 3.0	10.71 ± 2.8	3.558	2, 1261	**0.029**	−.0292	0.424	0.241	0.965	0.533	**0.032**
DUWAS work- compulsively (mean ± *SD*)	10.31 ± 2.7	9.66 ± 2.8	9.22 ± 2.4	9.654	2, 1261	**<0.001**	0.598	**0.004**	0.968	**<0.001**	0.370	0.171

*BW, back to work; HW, homeworking; WAFCS, work and family conflict scale; DUWAS, Dutch Work Addiction Scale; SD, standard deviation; df, degrees of freedom; crit.diff., critical difference.*

*Significant values (p < 0.05) are highlighted in bold.*

Independent-samples *t*-test showed at the uncorrected level a significant difference between women and men on the WAFCS work-family conflict score (*p* = 0.021), on the DUWAS Work Excessively score (*p* = 0.003), and on the DUWAS work compulsively score (*p* < 0.0001). Only the two DUWAS sub-scores differences survived the Bonferroni correction (*p* < 0.05/6 = 0.0083). There was no significant difference in Concern about BW and HW, and no significant difference in WAFCS family-work conflict scores emerged (as shown in Table 4).

**TABLE 4:** Questionnaires scores in Reopening, Second Wave, and Vaccination Round groups by gender.

	Reopening				Second Wave				Vaccination Round			
	
Variable	Females *N* = 451	Males *N* = 813			Females *N* = 319	Males *N* = 522			Females *N* = 213	Males *N* = 278		
	
	*M* ± *SD*	*M* ± *SD*	*t*-test	*p*	*M* ± *SD*	*M* ± *SD*	*t*-test	*p*	*M* ± *SD*	*M* ± *SD*	*t*-test	*p*
Concern about BW	2.89 ± 1.2	2.60 ± 1.2	3.920	0.683	3.00 ± 1.3	2.7 ± 1.3	3.243	0.819	2.83 ± 1.3	2.30 ± 1.2	4.622	<0.0001*
Concern about HW	1.85 ± 1.0	1.81 ± 1.0	0.718	0.665	1.90 ± 1.1	1.72 ± 0.9	2.581	0.155	1.80 ± 1.0	1.77 ± 1.0	0.353	0.724
WAFCS work-family conflict	14.29 ± 5.2	13.59 ± 5.1	2.332	0.021	13.21 ± 5.5	12.2 ± 5.3	2.665	0.008	13.69 ± 5.7	13.21 ± 5.3	0.968	0.334
WAFCS family-work conflict	8.24 ± 3.6	8.46 ± 3.8	−0.987	0.324	7.73 ± 3.4	7.75 ± 3.6	−0.075	0.940	7.9 ± 3.5	7.92 ± 3.2	−0.073	0.942
DUWAS work excessively	11.45 ± 2.9	10.94 ± 2.9	2.979	0.003*	11.51 ± 2.9	10.91 ± 2.9	2.936	0.003*	–	–	–	–
DUWAS work compulsively	10.1 ± 2.7	9.52 ± 2.7	0.515	<0.0001*	10.2 ± 2.8	9.58 ± 2.7	3.139	0.002*	–	–	–	–

*BW, back to work; HW, homeworking; WAFCS, work and family conflict scale; DUWAS, Dutch Work Addiction Scale; SD, standard deviation; df, degrees of freedom.*

*Significant values (p < 0.05) are highlighted in bold.*

### Concern About Back to Work and Homeworking, Work and Family Conflict Scale, and Dutch Work Addiction Scale Variables in the Second Wave Group by Gender and Age Classes

One-way ANCOVA showed a statistically significant difference in Concern about BW between age classes (*F*_2.838_, 4.909, *p* = 0.008) while controlling for gender; Bonferroni’s *post hoc* pointed out that a significant difference between under 35 and 36–54 groups (*p* = 0.006), stressing a higher level in under 35 subjects.

A statistically significant difference in WAFCS work-family conflict (*F*_2.838_, 6.829, *p* = 0.001) and WAFCS family-work conflict (*F*_2.838_, 7.766, *p* = <0.001) emerged between the three categories while controlling for covariate. Bonferroni’s *post hoc* test pointed out for WAFCS work-family conflict a significant difference, between 36–54 and over 55 groups (*p* < 0.001), stressing a higher level in 36–54 years old subjects. About WAFCS family-work conflict, the *post hoc* test highlighted a significant difference between under 35 and 36–54 groups (*p* < 0.001), pointing out a higher level in 36–54 years old subjects.

The one-way ANCOVA revealed a statistically significant difference in DUWAS work excessively score (*F*_2.838_, 3.330, *p* = 0.036) and DUWAS work-compulsively score (*F*_2,838_ = 8.009, *p* = <0.001) between the three age categories while controlling for gender. Bonferroni’s *post hoc* analyses highlighted no significant differences on the DUWAS work excessively score. Concerning the DUWAS work-compulsively score, the Bonferroni’s *post hoc* pointed out a significant difference between under 35 and over 55 groups (*p* < 0.001), and between 36–54 and over 55 groups (*p* = 0.040). These results pointed out a higher level in 35 years old subjects. No significant difference in Concern about HW emerged (as shown in Table 5).

**TABLE 5:** Questionnaires scores in the Second Wave group by age class.

Variable	<35 *N* = 204	36–54 *N* = 437	>55 *N* = 200	*F*	df	*P*	<35 vs. 36–54		<35 vs. >55		36–54 vs. >55	
									
							Crit. diff.	*p*	Crit. diff.	*p*	Crit. diff.	*p*
Concern about BW (mean ± *SD*)	3.06 ± 1.3	2,70 ± 1.3	2.80 ± 1.3	4.909	2, 838	**0.008**	0.341	**0.006**	0.189	0.449	−0.152	0.528
Concern about HW (mean ± *SD*)	1.77 ± 1.0	1,80 ± 0.9	1.77 ± 0.9	0.131	2, 838	0.878	–	–	–	–	–	–
WAFCS work-family (mean ± *SD*)	12.5 ± 5.5	13.19 ± 5.3	11.36 ± 5.1	6.829	2, 838	**0.001**	−0.758	0.278	0.913	0.270	1.671	**<0.001**
WAFCS family-work (mean ± *SD*)	7.05 ± 2.9	8.17 ± 3.8	7.51 ± 3.3	7.766	2, 838	**<0.001**	−1.117	**<0.001**	−0.448	0.616	0.669	0.080
DUWAS work excessively (mean ± *SD*)	11.39 ± 2.9	11.27 ± 2.9	10.59 ± 2.9	3.330	2, 838	**0.036**	0.088	1.000	0.672	0.067	0.583	0.060
DUWAS work compulsively (mean ± *SD*)	10.4 ± 2.7	9.84 ± 2.8	9.81 ± 2.8	8.009	2, 838	**<0.001**	0.523	0.073	1.109	**<0.001**	0.586	**0.040**

*BW, back to work; HW, homeworking; WAFCS, work and Family conflict scale; DUWAS, Dutch Work Addiction Scale; SD, standard deviation; df, degrees of freedom; crit.diff., critical difference.*

*Significant values (p < 0.05) are highlighted in bold.*

Independent-samples *t*-test showed a significant difference between women and men on the WAFCS work-family conflict score (*p* = 0.008), a result that, although not surviving the statistical correction, highlights a trend. In addition, analyses pointed out a significant difference surviving the Bonferroni correction in DUWAS work excessively score (*p* = 0.003) and DUWAS work compulsively score (*p* = 0.002), thus, showing higher scores for women in both cases. No significant difference in Concern about BW and HW or WAFCS family-work conflict emerged (as shown in Table 4).

### Concern About Back to Work and Homeworking, and Work and Family Conflict Scale Variables in the Vaccination Round Group by Gender and Age Classes

One-way ANCOVA highlighted no statistically significant difference in Concern about BW and HW, WAFCS work-family conflict, or WAFCS family-work conflict between age classes while controlling for gender (as shown in Table 6).

**TABLE 6:** Questionnaires scores in the Vaccination Round group by age class.

Variable	<35 *N* = 66	36–54 *N* = 316	>55 *N* = 109	*F*	df	*p*	<35 vs. 36–54		<35 vs. >55		36–54 vs. >55	
									
							Crit. diff.	*p*	Crit. diff.	*p*	Crit. diff.	*p*
Concern about BW (mean ± *SD*)	2.92 ± 1.3	2,50 ± 1.3	2.38 ± 1.1	2.913	2, 488	0.055	–	–	–	–	–	–
Concern about HW (mean ± *SD*)	1.76 ± 1.1	1,78 ± 1.0	1.82 ± 0.9	0.101	2, 488	0.904	–	–	–	–	–	–
WAFCS work-family (mean ± *SD*)	14.2 ± 5.8	13.48 ± 5.6	12.77 ± 5.1	1.249	2, 488	0.288	–	–	–	–	–	–
WAFCS family-work (mean ± *SD*)	7.33 ± 3.0	8.1 ± 3.6	7.73 ± 3.0	1.575	2, 488	0.208	–	–	–	–	–	–

*BW, back to work; HW, homeworking; WAFCS, work and Family conflict scale; DUWAS, Dutch Work Addiction Scale; SD, standard deviation; df, degrees of freedom; crit.diff., critical difference.*

Independent-sample *t*-test showed a significant difference in Concern about BW (*p* < 0.0001) between women and men, a result that survived the statistical correction. No significant difference in Concern about HW, WAFCS work-family conflict, and WAFCS family-work conflict scores emerged between women and men (as shown in Table 4).

### Correlates and Predictors

Table 7 shows all the results of correlational analyses significant at the uncorrected level *p* < 0.05 and the corrected level (*p* < 0.05/2 = 0.025).

**TABLE 7:** Demographic correlates of Concern about BW and HW, WAFC, and DUWAS in the three groups.

Variable	Reopening *N* = 1264		Second Wave *N* = 841		Vaccination Round *N* = 491	
			
	*r*	*p*	*R*	*p*	*r*	*p*
Concern about BW						
Number of children	−0.095	0.001*	−0.142	<0.0001*	−0.021	0.647
Number of HM days/week	0.183	<0.0001*	0.184	<0.0001*	0.349	<0.0001*
Concern about HW						
Number of children	0.011	0.701	−0.031	0.376	0.073	0.107
Number of HM days/week	−0.191	<0.0001*	−0.109	0.002*	−0.301	<0.0001*
WAFCS work-family conflict						
Number of children	0.046	0.104	0.023	0.507	0.053	0.242
Number of HM days/week	0.013	0.639	−0.022	0.519	−0.112	0.013*
WAFCS family-work conflict						
Number of children	0.150	<0.0001*	0.155	<0.0001*	0.032	0.486
Number of HM days/week	0.006	0.821	−0.032	0.349	−0.125	0.005*
DUWAS work excessively						
Number of children	0.012	0.665	−0.020	0.567	–	–
Number of HM days/week	−0.029	0.299	−0.090	0.009*	–	–
DUWAS work compulsively						
Number of children	−0.033	0.242	−0.049	0.153	–	–
Number of HM days/week	−0.036	0.198	−0.007	0.830	–	–

*BW, back to work; HW, homeworking; WAFCS, work and family conflict scale; DUWAS, Dutch Work Addiction Scale.*

*Significant values at uncorrected level (p < 0.05) are highlighted in bold.*

*Significant values at corrected level (p < 0.05/2 = 0.025).

In the Reopening group, the number of children was positively correlated to WAFCS family-work conflict score (*r* = 0.150, *p* < 0.0001) and negatively related to Concern about BW level (*r* = −0.095; *p* = 0.001). The number of days per week spent in HW was positively correlated to Concern about BW (*r* = 0.183, *p* < 0.0001) and negatively to Concern about HW (*r* = −0.191; *p* < 0.0001). All these results survived the Bonferroni correction.

In the Second Wave group, correlation analyses highlighted that the number of children was negatively related to the level of Concern about BW (*r* = −0.142, *p* < 0.0001) and positively to WAFCS family-work conflict score (*r* = 0.155; *p* < 0.0001). The number of days spent in HW per week showed a negative relationship with HWC (*r* = −0.109, *p* = 0.002) and with DUWAS work excessively sub-score (*r* = −0.090, *p* = 0.009). In contrast, a positive correlation with concern about BW score emerged (*r* = 0.184, *p* < 0.0001). All these results survived the statistical correction.

In the Vaccination Round group, correlation analyses pointed out a negative relationship between the number of days spent in HM per week and the level of Concern about BW (*r* = −0.301, *p* < 0.0001), WAFCS family-work conflict score (*r* = −0.125, *p* = 0.005), and WAFCS work-family conflict score (*r* = −0.112; *p* = 0.013). The number of HW days/week was also positively related to Concern about BW (*r* = 0.349, *p* < 0.0001). All these results survived the Bonferroni correction. No significant correlation concerning the number of children emerged.

A linear regression model was used to test if the number of children and the number of days per week spent in HW significantly predicted Concern about BW and HW, WAFCS indices, and Duwas work excessively sub-scale at each time point (as shown in Table 8).

**TABLE 8:** Predictors of Concern about BW and HW, WAFCS, and DUWAS variables in the three groups.

Predictors	Concern about BW														

	Reopening					Second Wave					Vaccination Round				
			
	*B*	95%CI	β	*T*	*p*	*B*	95%CI	β	*T*	*p*	*B*	95%CI	β	*T*	*p*
HM days/week	0.225	[0.157, 0.293]	0.179	6.502	<0.0001*	0.353	[0.221, 0.484]	0.177	5.256	<0.0001*	0.351	[0.267, 0.435]	0.349	8.202	<0.0001*
Number of children	−0.111	[−0.179, −0.044]	−0.089	−3.225	0.001*	−0.182	[−0.273, −0.091]	−0.133	−3.942	<0.0001*	0.006	[−0.104, 0.115]	0.004	0.1	0.920
	Concern about HW														
	T1					T2					T3				
	*B*	95%CI	β	*T*	*p*	*B*	95%CI	β	*t*	*p*	*B*	95%CI	β	*T*	*p*
HM days/week	−0.200	[−0.256, −0.143]	−0.19	−6.887	<0.0001*	−0.169	[−0.272, 0.066]	−0.111	−3.226	0.001*	−0.24	[−0.308, −0.171]	−0.297	−6.875	<0.0001*
Number of children	0.088	[−0.053, 0.061]	0.004	0.14	0.888	−0,038	[−0,109, 0.033]	−0.036	−1.052	0.293	0.054	[−0.035, 0.144]	0.052	1.194	0.233
	WAFCS work-family conflict														
	T1					T2					T3				
	*B*	95%CI	β	*T*	*p*	*B*	95%CI	β	*t*	*p*	*B*	95%CI	β	*T*	*p*
HM days/week	0.077	[−0.209, 0.363]	0.015	0.528	0.598	−0.173	[−0.726, 0.381]	−0.021	−0.613	0.540	−0.471	[−0.853, −0.088]	−0.109	−2.416	0.016*
Number of children	0.239	[−0.046, 0.524]	0.046	1.643	0.101	0.123	[−0.259, 0.504]	0.022	0.631	0.528	0.255	[−0.245, 0.755]	0.045	1.001	0.317
	WAFCS family-work conflict														
	T1					T2					T3				
	*B*	95%CI	β	*T*	*p*	*B*	95%CI	β	*t*	*p*	*B*	95%CI	β	*T*	*p*
HM days/week	0.045	[−0.163, 0.253]	0.012	0.425	0.671	−0.131	[−0.490, 0.227]	−0.025	−0.719	0.472	−0.330	[−0.567, 0.094]	−0.124	−2.745	0.006*
Number of children	0.569	[0.362, 0.777]	0.150	5.393	<0.0001*	0.565	[0.318, 0.811]	0.153	4.488	<0.0001*	0.079	[−0.230, 0.388]	0.023	0.504	0.614
	DUWAS work excessively														
	T1					T2					T3				
	*B*	95%CI	β	*T*	*p*	*B*	95%CI	β	*t*	*p*	*B*	95%CI	β	*t*	*p*
HM days/week	−0.08	[−0.248, 0.078]	−0.029	−1.023	0.307	−0.405	[−0.705, −0.105]	−0.091	−2.652	0.008*	−	−	−	−	−
Number of children	0.033	[−0.130, 0.195]	0.011	0.396	0.692	−0.074	[−0.281, 0.132]	−0.024	−0.708	0.479	−	−	−	−	−

*BW, back to work; HW, homeworking; WAFCS, Work and Family conflict scale; DUWAS, Dutch Work Addiction Scale.*

*Significant values at uncorrected level (p < 0.05) are highlighted in bold.*

*Significant values at corrected level (p < 0.05/2 = 0.025).

In the Reopening group, the overall regression was statistically significant for Concern about BW (*R*^2^ = 0.041, *F*_2.1261_, 27.137, *p* < 0.0001), Concern about HW (*R*^2^ = 0.036, *F*_2.1261_, 23.788, *p* < 0.0001), and WAFCS family-work conflict (*R*^2^ = 0.023, *F*_2.1261_, 14.567, *p* < 0.0001). The number of days per week spent in HW significantly predicted the level of Concern about BW (β = 0.179, *p* < 0.0001) and the level of Concern about HW (β = −0.19, *p* < 0.0001). The number of children significantly predicted the level of concern about BW (β = −0.089, *p* = 0.001) and WAFCS family-work conflict score (β = 0.150, *p* < 0.0001).

In the Second Wave group, the overall regression was statistically significant for the level of concern about BW (*R*^2^ = 0.051, *F*_2_._838_, 22.69, *p* < 0.0001), the level of Concern about HW (*R*^2^ = 0.013, *F*_2_._838_, 5.599, *p* = 0.004), WAFCS family-work conflict (*R*^2^ = 0.24, *F*_2_._838_, 10.520, *p* < 0.0001), and DUWAS work excessively score (*R*^2^ = 0.09, *F*_2_._838_ = 3.682, *p* = 0.026). The number of days spent in HW per week significantly predicted the level of Concern about BW (β = 0.177, *p* < 0.0001), the level of concern about HW (β = −0.111, *p* = 0.001), and the DUWAS work excessively score (β = −0.091, *p* = 0.008). The number of children significantly predicted the level of Concern about BW (β = −0.133, *p* < 0.0001) and the WAFCS family-work conflict score (β = 0.153, *p* < 0.0001).

In the Vaccination Round group, the overall regression was statistically significant for the level of concern about BW (*R*^2^ = 0.122, *F*_2_._488_, 33.759, *p* < 0.0001), the level of concern about HW (*R*^2^ = 0.093, *F*_2_._488_, 25.057, *p* < 0.0001), WAFCS work-family conflict (*R*^2^ = 0.015, *F*_2_._488_, 3.610, *p* = 0.028), and WAFCS family-work conflict (*R*^2^ = 0.016, *F*_2_._488_, 4.015, *p* = 0.019). The number of days per week spent in HW significantly predicted the level of concern about BW (β = 0.349, *p* < 0.0001), the level of concern about HW (β = −0.297, *p* < 0.0001), WAFCS work-family conflict (β = −0.109, *p* = 0.016), and WAFCS family-work conflict (β = −0.124, *p* = 0.006). The number of children did not predict any variable.

## Discussion

The primary purpose of this study was to provide insights into office workers’ adjustment during the first COVID-19 pandemic year in an Italian sample of employees of a large banking group. In particular, we aimed to highlight different possible reactions in workaholism, work-family conflict, and Concern about BW in three different stages of the pandemic, namely at the Reopening after the end of the first lockdown (March 2020), the Second Wave (October and November 2020), and the first Vaccination Round (May and June 2021). As a secondary purpose, we aimed at investigating the role of socio-demographic variables, specifically gender, age, number of children in the family, and days spent in HW per week, as the related factors of adaptation and Concern about BW. Three main results emerged. First, different adaptive attitudes and behavioral reactions characterized the three pandemic stages. As hypothesized, Reopening was characterized by the highest levels of family-work conflict. Second, as hypothesized, women and younger subjects had the worse adjustment outcome during HW and showed higher skepticism to recent BW. On the contrary, older workers showed the best adaptation throughout the three pandemic stages. Third, the number of days spent in HW and the number of children were predictors of Concern about BW and work-family conflicting overlap.

### Different Attitudes in Different Pandemic Stages

Concerning our first result, Reopening and, although, to a lesser extent, Vaccination Round were characterized by the harshest overlap between family and work-life dimensions. Conversely, the Second Wave did not imply significant changes. These results suggest that the Reopening and Vaccination Round stages were most critical for workers as they were characterized by changing rules related to socio-sanitary conditions. Thus, individuals in both periods had to undergo new demanding work and family adjustments. Actually, in Reopening, HW, although possibly alternating with work at the office, was still a novelty or even a hazard for most employees and most organizations, thus, representing a relevant source of stress (Hecker, 2020; Fukumura et al., 2021; Hamouche, 2021). Homeworkers not only have experienced an abrupt overlap between work and family lives (Moretti et al., 2020) but also had to challenge a new working strategy, for which workers and organizations were largely unprepared (Söderbacka et al., 2020; Shao et al., 2021; Xiao et al., 2021), so that most individuals were caught off guard and had to adjust by their means (Guler et al., 2021).

In the Second Wave stage, work-family conflict was the lowest. This result lets us speculate that HW was the new normality in those months, so no specific adaptation demand for working activity or family life was required. However, the Second Wave was the period of higher Concern about HW, analogously, although to a lesser extent, to Vaccination Round. This evidence may be accounted for the prolonged state of social and economic uncertainty that could be less impactful at the Reopening stage, which, although prematurely, seemed to prelude that the end was near and could thus be more biased by hope. To note, the levels of Concern about BW were higher than the Concern about HW regardless of the pandemic stage. It is plausible that workers were more concerned about being infected and thus more apprehensive about leaving their homes (Zhang J. et al., 2020). Moreover, most of them might have been quarantined, infected, or indirectly suffered from this virus (Brooks et al., 2020), suggesting that they were more psychologically vulnerable (Shaw et al., 2020).

An analogous stressful trend was observed in the Vaccination Round that was characterized by higher family-work conflicting overlap. Vaccination Round was a period of changes, too, as the vaccination campaign encouraged the back to the office. Despite the apparent positive news and the hope represented by medical and scientific progression in the battle against COVID-19, after a long period of adjustment and a gained balance in work activity and family needs, getting back to work may have been experienced as upsetting again. Thus, although a necessity, returning to work can be a source of discomfort and anxiety for employees (Hamouche, 2021). During the Vaccination Round, the Concern about BW was lower than in the two previous pandemic stages. This apparent paradox in the Vaccination Round can be accounted for by the contemporary presence of a sense of hope and safety, which is supposed to encourage a return to pre-pandemic everyday life and work newly perceived as interfering with family life. Indeed, these results highlight the stressful effects on employees due to sudden adaptive requirements in family and work organizations.

### The Role of Gender and Age

Concerning our second result, women and men have challenged the three pandemic stages differently, with a worse outcome for women, especially in the Reopening and the Second Wave. First, women reported higher perceived interference of work on family needs. The differences in psychological impacts may reflect traditional gender roles (Oakman et al., 2020). Indeed, in Italy, the burden of domestic activities is still borne mainly by women, who dedicate a larger share of their unpaid working time to household activities than men (ISTAT, 2019). Far from being a trivial issue, this evidence reflects how people, not only in Italy, differently stigmatize the flexible working of men and women. Several studies have shown that when women take up smart working, especially HW, they are expected to carry out domestic work simultaneously while working (Chung and van der Lippe, 2020). Given these premises, our data contribute to casting light on the Italian social and labor context in the COVID-19 pandemic era, not only because of the markedly strict lockdown measures taken to contain the crisis but also from a gender standpoint, as Italy is characterized by both by traditionally high gender gaps in the labor market and conservative gender roles (Del Boca et al., 2020). Not surprisingly, an abrupt increase in family demands in the initial stages of the pandemic (e.g., taking care of children due to school closure) may have severely interfered with working at home. Our data are consistent with studies on psychological symptoms during the first stages of the pandemic in Italy. They describe a higher level of distress, sleep disorders, and anxiety than men, even though this gap in the mid-term tended to reduce (Salfi et al., 2020).

Interestingly, this is evident during the Reopening and the Second Wave, while in the Vaccination Round stage, women are less concerned with the overlap between work and private life but show more significant concern for coming back to the workplace. This result supports the hypothesis that after a long and stressful period of work-family routine adjustment, women best appreciate HW’s advantages and are less prone to BW (Fukumura et al., 2021). It is plausible that adapting to HW may be associated with increased free time and potential scope for improved work-life balance (McDowell et al., 2021). However, it is true that women during the Reopening and the Second Wave showed a higher propensity for workaholism. Previous studies pointed out that female workers demonstrated higher productivity than male workers (Awada et al., 2021). However, our data suggest that for women, the risk to shift from efficient work engagement to workaholism during the Reopening and the Second Wave was higher than for men.

Regarding age, the youngest subjects were the most worried about BW and were more vulnerable to workaholism, particularly compulsive overworking, regardless of the pandemic stage. Most studies from Italy, as well as from other eastern and western countries, highlighted that lower age represents a risk factor for developing psychological symptoms due to the impact of the pandemic (Gualano et al., 2020; Prati, 2020; Amicucci et al., 2021; Marelli et al., 2021). Additionally, they integrate them shedding light on the working dimension. In younger subjects, excessive engagement in working activities can be accounted for a higher achievement orientation and desire for self-esteem (Ng et al., 2007). Indeed, the forced sudden HW organization has abruptly pushed employees to re-arrange behaviors, habits, and communication styles. Several structural, logistic, and technological inconveniences may have caused a longer time to work and greater involvement in job-related tasks. Moreover, as a more future-oriented attitude characterizes young adults, they may also be more prone to underestimate the pandemic’s lasting effects and expect a closer conclusion of the emergency and a fast return to “normality” (Ernst and D’Argembeau, 2017). There was an expectation that risks were giving place in younger populations to delusion and frustration with the possible protraction of pandemic threat. Notably, our data let us speculate that this adjustment effort jeopardized subjects with lower working experience, while older office workers could count on their more comprehensive expertise. In addition to this, in literature, older adults are frequently described as characterized by efficient emotional regulation strategies that focus on positive emotions and reduce negative affect (Scheibe and Carstensen, 2010). This attitude was previously described during the crisis outbreak too (Ceccato et al., 2020; Gualano et al., 2020; Jiang, 2020; Lopez et al., 2020; Prati, 2020). In addition to this, the over 55 years old segment of the sample was the least worried about BW, evidence which can be traced back to the fact that in Italy, the older subjects had priority in vaccination, thus, possibly contributing to self-safety sensation. The more senior employees’ favorable attitude toward BW can also be accounted for the difficulties these employees may have with technological tools and their potential less ability to adapt to changes, mainly if they occur quickly (Galanti et al., 2021). Finally, it is worthy of note that the middle age group (36–54 years old) was the most jeopardized by the work-private life overlap. In particular, the most suffered the interference of family on working activities. This evidence may suggest that these workers had more difficulties in managing the work-life balance due to greater involvement in career achievements and greater family responsibilities at the same time.

### Predictors of Concern About Homeworking and Back to Work

About our third result, on one side, the higher number of days per week spent in HW was related to lower Concern about HW and higher Concern about BW, thus, confirming the skepticism to modify the new organization of everyday routine and the recently gained balance. Those who spent more days in HW also had a lower family-work conflict and a lower tendency to work excessively. In this sense, our data showed the perceived advantages of HW and the reluctance to give them up. Interestingly, our results highlighted that the individual family status appears likely to disparately affect how COVID-19 impacts individuals’ life and work (Kniffin et al., 2021). A higher number of children were related to the lower Concern about BW and a higher level of work-family conflict. Thus, those workers who had more children seem favorable to returning to the workplace, possibly because they perceive family interference in working activities in HW as harsher.

### Limitations

Some limitations must be considered when interpreting the results of the present study. First, our main concern is selection bias. As we recruited only employees of a single Italian banking group, results may not represent the general Italian employee population, and we do not know whether the observed data are strictly related to professional groups. Moreover, those who have accepted our invitation to participate in the study as volunteers may also be more engaged with the topic and more sensitive toward psychological issues. Second, although the panel invited was the same for the three surveys, since participation was completely voluntary and anonymous, we cannot guarantee that the three samples included the same subjects. Thus, we cannot exclude those changes in attitudes that would be partly due to the involvement of different subjects each time. For the same reason, our study cannot be defined strictly as a longitudinal design but rather a three-survey case study. Nonetheless, our data cast light on possible changes in behaviors and attitudes related to different time stages in samples drawn from the same circumscribed population. However, future works underpinned by actual longitudinal data will fill this gap. Third, the items to investigate the Concern about BW and HW were single-question items. Thus, possibly they do not fully catch the multifaceted phenomenon object of the study. Future studies deepening this issue with more articulated tools are desirable. Fourth, data on workaholism were reliable only in the Reopening and Second Wave; thus, we have no comparable data for the Vaccination Round stage, determining an information gap. Future studies investigating the workaholism also in more recent pandemic stages are required. Fifth, our samples included bank employees who were relatively safe from negative economic consequences due to the pandemic impact and continued to work even during the lockdown. This condition might represent a protective factor against the harsher forms of stress affecting mental health. For instance, previous studies showed that people who stopped working or whose job position was threatened reported worse mental and physical health conditions, distress, and sleep disturbances during the current pandemic emergency as compared with those who worked at home (Zhang S.X. et al., 2020; Salfi et al., 2021b). Regarding this last point, we did not collect data on possible sleep disorders due to changes in daily time schedules related to the reorganization of individual and family routines in HW. Given the salience of the issue for psychological wellbeing, a deepening by further studies is required. Finally, we have performed primarily descriptive and correlational analyses. Indeed, the number of measures, data, and subjects would have allowed more complex and potentially fruitful statistical analyses. Nonetheless, given the study’s aims, the characteristics of the samples, and the state-of-the-art on the topic, an observational approach proved to be more appropriate, as indeed in previous studies (Giedrė Raišienė et al., 2020; Donati et al., 2021). Additional studies are required to deepen these issues beyond the descriptive/observational approach.

### Conclusion

To the best of our knowledge, our study is one of the first to show long-term perspective data on adjustment to work during the pandemic of COVID-19 in a stress theoretical framework. Changes in the job context during the COVID-19 pandemic are highly likely to be perceived as severely stressful by the employees. They repeatedly required an abrupt reorganization of one’s work and family life (Holmes and Rahe, 1967), workers had very low control and choice latitude on these events (Karasek et al., 1981), and one’s primary work and life goals may have been upset or even threatened by incoming job demands (Carver and Scheier, 1999). Our results support the hypothesis that the pandemic stages where adaptation to a new everyday life routine is abruptly and repeatedly compelled are more stressful and exacerbate work-family conflict and commitment to work. Frequent and heavily demanding changes are expensive for the individuals, and when workers reach a balance, they are reluctant to give the new equilibrium up, thus, resisting the new organizational proposals. Adjustment to work routine and modalities is a complex process that implies several critical aspects, such as satisfaction, commitment, productivity, and the ability to balance work and non-work demands, as also stressed in the theory of work adjustment (Dawis, 2005; van Zoonen et al., 2021). Notably, stress due to work and family life adaptation affects the wellbeing of office workers regardless of the improvements in health conditions. At the same time, a relatively stable and foreseeable organizational framework is related to the lower levels of work-family conflict even when sanitary conditions are worsening. Not to say, self-safety perception, the need for social distancing, wearing masks, and additional preventive measures in the workplace can discourage office workers from regularly going to the workplace (Hamouche, 2021). When a voluntary option, HW is perceived as the new normal and as advantageous, especially by women and younger subjects (Shepherd-Banigan et al., 2016), with data consistent with previous cross-sectional studies (Guler et al., 2021). This evidence is valid more generally for smart or flexible working, defined as a new way to organize work that includes the flexibility of location (working from home, but also a different location than the usual workplace) and flexibility of time (a personalized work schedule). This new way of working can entail employees’ control over when or where they work (Chung and van der Lippe, 2020; Toscano and Zappalà, 2020). Consequently, organizations should encourage a cultural and organizational configuration of working activities (Iannotta et al., 2020) to understand and interpret a new way of working. Moreover, a road map for BW after such a long period of HW (Rueda-Garrido et al., 2020; Hamouche, 2021) is strongly required to help reduce their level of stress and prevent the risk of mental health issues. Moreover, organizations should consider the complex intersections of work-life and home-life to develop supportive policies and resources. The new way of working trajectories would imply the crucial aim to create and implement best practices for working from home to maintain a good level of productivity, achieve the right level of work and life balance, and maintain a good level of physical and mental health (Awada et al., 2021; Magnavita et al., 2021; Okuyan and Begen, 2021).

## Data Availability Statement

The raw data supporting the conclusions of this article will be made available by the authors, without undue reservation.

## Ethics Statement

The studies involving human participants were reviewed and approved by Joint Ethical Committee for Research of Scuola Normale Superiore, Scuola Superiore Sant’Anna, and IMT School for Advanced Studies Lucca. The patients/participants provided their written informed consent to participate in this study.

## Author Contributions

MO: conception and design of the work, data collection, data interpretation, and drafting the article. DP: data analysis and data interpretation. SD’A, FM, and DR: conception and design of the work. NL: critical revision of the article. AM: coordination of research activity, conception and design of the work, and critical revision of the article. ER: responsibility for supervising research, funding acquisition, critical revision of the article, and final approval of the version to be published. All authors contributed to the article and approved the submitted version.

## Conflict of Interest

SD’A and FM were employed by the company Intesa Sanpaolo Innovation Center S.p.A. DR was employed by the company Intesa Sanpaolo DC Tutela Aziendale - Sicurezza sul Lavoro ed Ambiente. The remaining authors declare that the research was conducted in the absence of any commercial or financial relationships that could be construed as a potential conflict of interest.

## Publisher’s Note

All claims expressed in this article are solely those of the authors and do not necessarily represent those of their affiliated organizations, or those of the publisher, the editors and the reviewers. Any product that may be evaluated in this article, or claim that may be made by its manufacturer, is not guaranteed or endorsed by the publisher.
